# Metallomic profiles of pregnant women living with obesity in the UK: a secondary analysis of UPBEAT

**DOI:** 10.1093/mtomcs/mfaf031

**Published:** 2025-08-12

**Authors:** João Agostinho de Sousa, Alexander Griffiths, Kathryn V Dalrymple, Sara L White, Ferdinand von Meyenn, Lucilla Poston, Jessica Rigutto-Farebrother, Angela C Flynn

**Affiliations:** Laboratory of Nutrition and Metabolic Epigenetics, Department of Health Sciences and Technology, ETH Zurich, Zürich 8092, Switzerland; PSI Center for Scientific Computing, Theory and Data, Paul Scherrer Institute, Villigen 5232, Switzerland; London Metallomics Facility, King’s College London, London SE1 9NH, UK; Department of Nutritional Sciences, School of Life Course and Population Sciences, King’s College London, London SE1 8WA, UK; Department of Women and Children’s Health, School of Life Course and Population Sciences, King’s College London, London SE1 7EH, UK; Laboratory of Nutrition and Metabolic Epigenetics, Department of Health Sciences and Technology, ETH Zurich, Zürich 8092, Switzerland; Department of Medical & Molecular Genetics, School of Basic & Medical Biosciences, King’s College London, London SE1 9RT, UK; Department of Women and Children’s Health, School of Life Course and Population Sciences, King’s College London, London SE1 7EH, UK; Laboratory of Nutrition and Metabolic Epigenetics, Department of Health Sciences and Technology, ETH Zurich, Zürich 8092, Switzerland; Global Center for the Development of the Whole Child, University of Notre Dame, Notre Dame, IN 46556 United States; Department of Nutritional Sciences, School of Life Course and Population Sciences, King’s College London, London SE1 8WA, UK; School of Population Health, Royal College of Surgeons in Ireland, Dublin 2, D02 DH60 Ireland

## Abstract

Characterization of serum metal element concentrations in pregnancy enables the elucidation of relationships with maternal-fetal and neonatal health. Metal elements in the blood serve as essential cofactors for enzymatic reactions and contribute to blood gas homeostasis, hormone synthesis, and physiological immune function for mother and fetus. Sub-optimal concentrations of some metals have been linked to adverse outcomes, including preterm birth, low birth weight, and impaired neurodevelopment. Maternal obesity also adversely influences metabolic status, including metal metabolism, with the potential for a heightened risk of complications at delivery and long-term health issues in offspring. Research on metal element levels in pregnant women with obesity and their effects on pregnancy outcomes is however limited. This study aims to characterize mid-gestation serum concentrations of 18 metal elements in samples from 755 pregnant women with obesity enrolled in the UK Pregnancies Better Eating and Activity Trial (UPBEAT) and identify associations with pregnancy outcomes. We found that calcium concentration tended to decrease with increasing parity, with an estimated reduction of 6.03 mg/L in multiparous participants compared to nulliparous participants (95% CI: −9.50 to −2.57 mg/L, *P =* 0.001). Additionally, elevated manganese concentrations at mid-pregnancy were associated with an increased incidence of antepartum haemorrhage after 34 weeks (OR: 4.62, 95% CI: 2.06–12.4, *P <* 0.001), and higher maternal phosphorus levels were linked to neonatal intensive care unit admissions (OR: 2.83, 95% CI: 1.75–4.67, *P <* 0.001). A future focus on dysregulation of these metal elements is needed to improve understanding of the clinical associations observed.

## Graphical Abstract

**Graphical Abstract:**
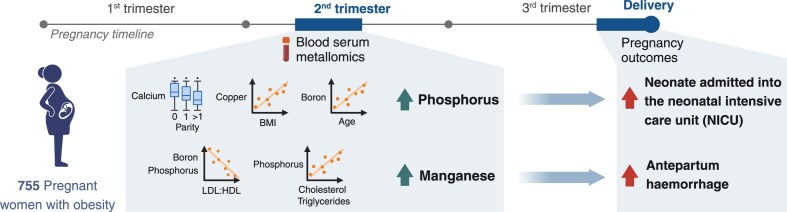
Serum metallomic profiles in mid-pregnancy link maternal and neonatal outcomes in women with obesity.

## Introduction

Metal elements are quintessential to human life. Physiological roles include contributions to oxygen transport and energy metabolism, immune responses, cell division, and antioxidant defence. Metal elements also act as critical enzyme cofactors and are integral to metalloproteins that comprise 30%–40% of all proteins in the body [1–4]. Despite this, they are often lacking in the diet. The United Kingdom National Diet and Nutrition Survey 2016–2019 found low intakes of some metals in up to almost half of a population sample of adult women aged 19–64 years. Twenty-five % had iron (Fe) intake below the UK lower reference nutrient intake (LNRI). Potassium (K) was low in 24%, magnesium (Mg) and zinc (Zn) in 11% and 7%, respectively, and 46% of the sample had intakes of selenium (Se) below the LNRI [5]. This is particularly concerning, since a proportion of these women are of reproductive age and pregnancy may increase micronutrient requirements. Deficiencies may also be exacerbated [6], putting both maternal and fetal/neonatal health at risk.

In addition, exposure to toxic metals during pregnancy can be deleterious to maternal and offspring outcomes. Several trace elements, including lead (Pb), arsenic (As), cadmium (Cd), and mercury (Hg), have been associated with adverse gestational consequences such as pre-eclampsia, low birth weight, and preterm birth [7]. Exposure to and accumulation of toxic metals in infants and children may impede normal growth and development, from effects of the toxic metals themselves and due to essential mineral deficiencies, and associations between maternal and offspring toxic metal profiles have been previously reported [8].

Obesity is a physiological state that puts micronutrient status at risk [9, 10]. Impaired metal homeostasis can have consequences for the function of metabolic pathways, which may, in turn, contribute to the development of obesity and other related comorbidities [11]. Obesity is generally associated with deficiencies in Mg, Zn, Fe, and Se, and with elevated copper (Cu) levels [11]. Furthermore, epidemiological studies have demonstrated an association between exposure to heavy metals and incidence of obesity and metabolic syndrome [12].

Accurate assessment of essential and non-essential trace metals in human serum can be achieved using metallomic analyses. Metallomics is a relatively new and fast-growing domain that has considerable potential to inform public health initiatives and policy by providing information on essential metal intakes as well as exposure to toxic metal elements. Metallomics profiles in certain morbidities during pregnancy, e.g. gestational diabetes [13–15], have now been described, although there is only sparse data from at-risk groups including pregnant women living with obesity.

To address this gap, we have profiled 18 metal elements in the blood of pregnant women with obesity. Although not all measured elements are strictly metals, we use the term ‘metal elements’ throughout for consistency and brevity. The specific aim of these exploratory analyses was to use metallomics to generate an early pregnancy ‘metallo-profile’ in a cohort of diverse inner-city women living with obesity.

## Results

### Characteristics of the study population

**Figure 1.**
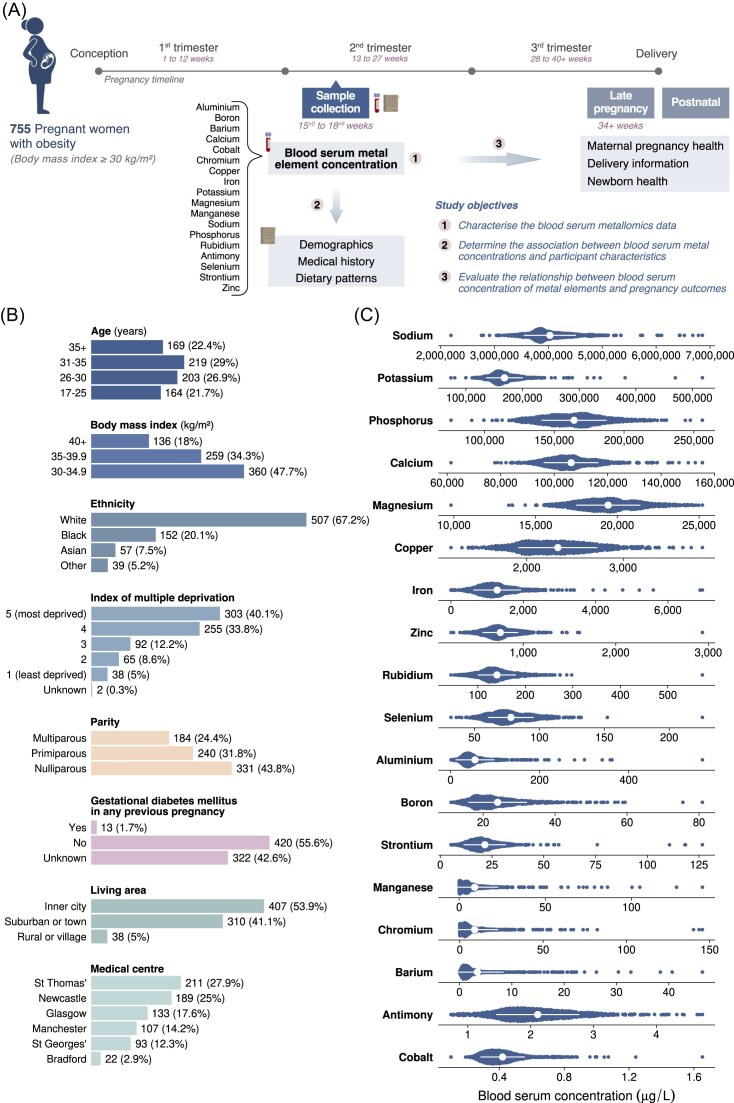
Graphical representation illustrating the study design and objectives, accompanied by the characterization of the participants’ data. (A) Study overview and objectives. (B) Participant distribution based on demographic and medical history variables, including age, BMI, ethnicity, index of multiple deprivation, parity, history of GDM in any previous pregnancy, living area, and the medical centre in which participants were approached. (C) Distribution of metal element concentrations in the serum across participants. Each data point represents the serum concentration of an individual participant. After evaluating the need for gestational age adjustment, only unadjusted results are reported.

Of the 1555 pregnant women who took part in the UPBEAT study, 755 who provided serum samples in the second trimester of pregnancy, and for which the data passed quality control requirements during the determination of element concentrations and data preparation (see Methods), were included in this analysis (Fig. 1A). The characteristics of the participants are presented in Table S1 and Fig. 1B. The median (IQR) participant age was 31 (26–35) years, and median (IQR) BMI was 35.3 (32.8–38.7) kg/m^2^, with 47.7% participants having a BMI between 30 and 34.9 kg/m^2^. The majority of participants were White (67.2%), followed by Black (20.1%), Asian (7.5%), and other (5.2%) ethnic groups. Forty % of participants lived in the most deprived area category and resided in either inner city (53.9%) or suburban areas (41.1%). Over half of participants were multiparous (56.2%).

### Metal concentrations in serum samples

**Figure 2.**
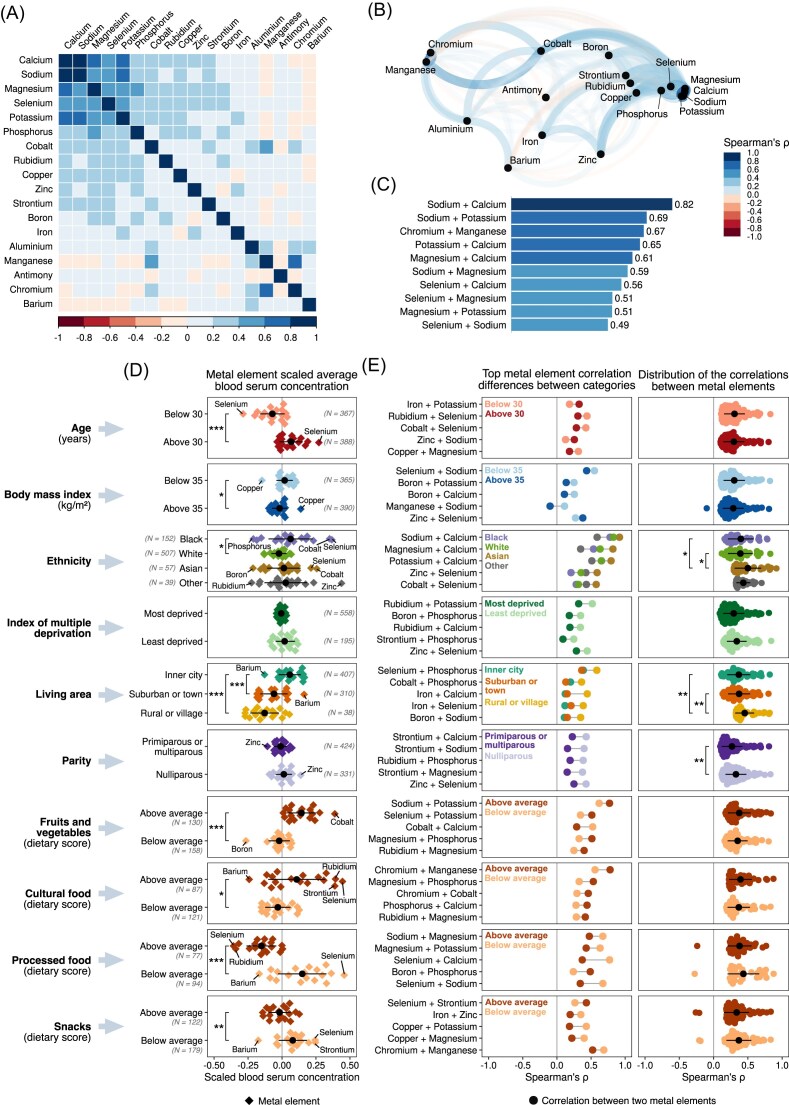
Characterization of metal element serum concentrations. (A) Spearman’s correlation between the metal elements’ serum concentrations. (B) Network representation displaying element correlations, with a closer graphical proximity indicating higher correlation in serum concentrations between metal elements. (C) Top 10 results of the correlation between pairs of elements. (D) Distribution of average scaled serum concentration by variable and categorical level. Each data point represents an element, with blood concentration scaled to centre around the average and expressed in standard deviations. (E) On the left, top 5 cross-correlations between elements selected based on their significant correlation differences between the categorical levels defined in (D). On the right, distribution of all significant cross-correlations between elements for the same categories.

The serum metal element concentrations in the second trimester of pregnancy are shown in Fig. 1C and Fig. S1. Sodium (Na) had the highest mean concentration of 4027.73 mg/L (175.20 mmol/L), followed by potassium (K), phosphorus (P), and calcium (Ca) (168.05 mg/L or 4.30 mmol/L; 164.19 mg/L or 5.30 mmol/L; and 106.37 mg/L or 2.65 mmol/L, respectively). Cobalt (Co) had the lowest mean serum concentration at 0.420 µg/L. Notable correlations between metals are found in Fig. 2A–C and Table S2.

### Association between serum metal concentrations and maternal characteristics

There were several associations between metal concentrations and maternal characteristics. The serum concentration of several metals increased with maternal age, with Se being higher in older participants (≤30 years old: 73.09 µg/L; ≥30 years old: 82.86 µg/L, *P <* 0.001). Participants with a BMI above 35 kg/m² had lower serum metal concentrations than women with a BMI < 35 kg/m^2^ except for copper (Cu), which was higher in this group (≥35 kg/m²: 2378.56 µg/L or 37.43 µmol/L; ≤35 kg/m²: 2262.01 µg/L or 35.60 µmol/L, *P <* 0.001). Metal concentrations varied with ethnicity, with Ca, Co, Cu, rubidium (Rb), Se, strontium (Sr) being higher in Black compared to White participants (*P <* 0.05), as well as geographical setting, with Ca, Co, Mg, Rb, Se, Na, Sr, being higher in participants living in inner-city areas (*P <* 0.05). Moreover, participants with dietary patterns characterized by fruits, vegetables, and cultural foods had higher overall metal concentrations, with Se being particularly elevated in those consuming more fruits and vegetables, and Rb higher in those consuming more cultural foods (*P <* 0.05). Conversely, participants with an above-average intake of processed foods and snacks had lower overall metal concentrations when comparing with participants with a below-average intake, notably lower levels of Rb and Co were observed in participants with above-average intake of processed foods (*P <* 0.05; Fig. 2D and Tables S2–5).

When assessing the associations between pairs of metal elements and maternal characteristics, notable interactions were observed, particularly for Ca and Mg (overall Spearman’s rho = 0.61). The correlation between Ca and Mg varied across different ethnicities, with Spearman’s rho ranging from 0.35 to 0.82. Participants with dietary patterns characterized by processed foods showed a lower correlation between Ca and Se (overall Spearman’s rho = 0.56) and Na and Se (overall Spearman’s rho = 0.49). Additionally, participants living in rural areas showed a stronger correlation between Ca and iron (Fe) (Spearman’s rho = 0.44) compared to those living in suburban areas (Spearman’s rho = 0.15) or in inner-city urban areas (Spearman’s rho = 0.12; Fig. 2E and Tables S6 and S7).

### Adjusted regression analyses

**Figure 3.**
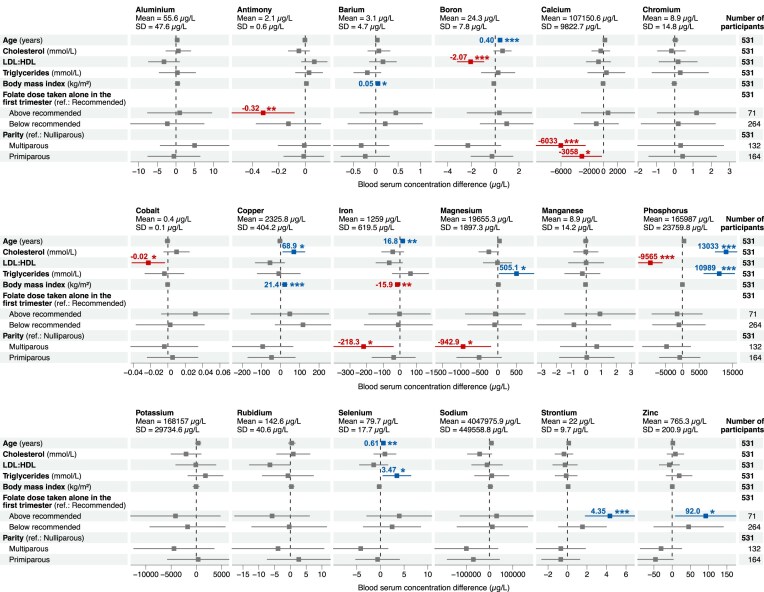
Results from the median regression analysis examining the relationship between participant characteristics and serum element concentrations between 15 ^+^  ^0^ and 18^+6^ weeks’ gestation. Each column in the figure table presents regression results for a specific element, where the element serum concentration served as the dependent variable. Each row presents the estimates and 95% confidence intervals for each independent variable. the models were built using 37 independent variables, and only those displaying a *P*-value below 0.001, or at least one of its categorical levels, are depicted in this figure. For a comprehensive overview, please refer to Table S9 for the complete results.

The previous analyses aimed to identify overall metal concentration patterns across participant characteristics but did not adjust for them. To assess associations between metal concentrations and maternal characteristics, we performed regression analyses by adjusting for 37 participant characteristics (Fig. S2 and Table S8). Of note, we found that serum concentrations of boron (B), Fe, and Se were positively associated with age, with changes of 0.40 (95% CI: 0.24–0.57, *P <* 0.001), 16.82 (95% CI: 4.21–29.43, *P =* 0.009), and 0.61 µg/L (95% CI: 0.22–0.99, *P =* 0.002) per year, respectively. Se levels were also higher among participants with dietary patterns characterized by a higher intake of cultural foods (3.02 µg/L, 95% CI: 0.85–5.19, *P =* 0.006) and lower for those with A level education when compared with participants with a degree (−8.09 µg/L, 95% CI: −13.38 to −2.80, *P =* 0.003). Fe concentration was negatively associated with BMI (−15.86 µg/L, 95% CI: −25.53 to −6.20, *P =* 0.001), while copper (Cu) and barium (Ba) were positively associated with BMI, increasing by 21.40 (95% CI: 9.11–33.69, *P <* 0.001) and 0.05 µg/L (95% CI: 0.01–0.10, *P =* 0.027) per unit of BMI, respectively. Ca levels were inversely associated with the number of pregnancies, with decreases of −3057.84 µg/L (95% CI: −5840.42 to −275.26, *P =* 0.031) in nulliparous and −6032.62 µg/L (95% CI: −9496.23 to −2569.01, *P <* 0.001) in multiparous participants. Similarly, Fe and Mg concentrations were lower in multiparous participants compared to nulliparous ones (Fe: −218.29 µg/L, 95% CI: −396.58 to −39.99, *P =* 0.017; Mg: −942.89 µg/L, 95% CI: −1690.91 to −194.88, *P =* 0.014). P concentrations were positively associated with total cholesterol and triglycerides (13 032.52 µg/L, 95% CI: 9819.25–16 245.79, *P <* 0.001; 10 989.32 µg/L, 95% CI: 6416.38–15 562.26, *P <* 0.001 per mmol/L, respectively) and negatively associated with the LDL to HDL ratio (−9564.74 µg/L, 95% CI: −13 102.05 to −6027.44, *P <* 0.001). B concentrations were also negatively associated with the LDL to HDL ratio (−2.07 µg/L, 95% CI: −3.19 to −0.96, *P <* 0.001). For complete results, see Figs 3 and S3, and Table S9.

### Association between serum metal concentrations and pregnancy outcomes

**Figure 4.**
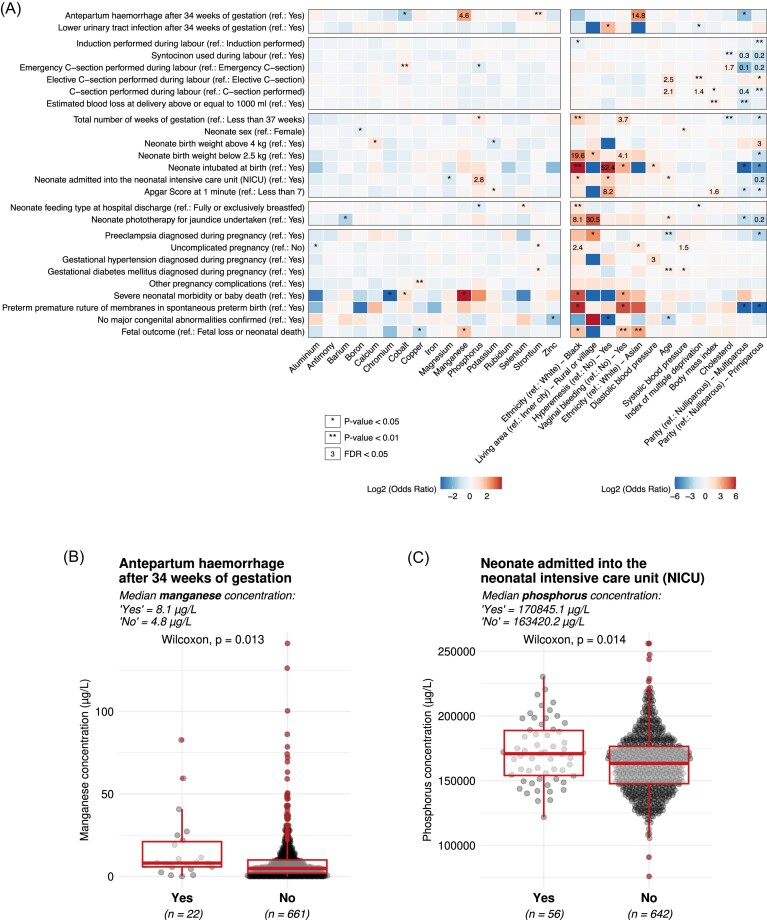
Serum metal element concentrations and their associations with pregnancy outcomes. (A) Overview of metal elements and participant information variables showing statistically significant associations with pregnancy outcomes, as identified in logistic regression analyses (*P < 0.05; **P < 0.01; Odds ratio value FDR < 0.05). (B) Manganese serum concentration distributions measured between 15^+0^ and 18^+6^ weeks’ gestation, categorized by the pregnancy outcome variable ‘Antepartum haemorrhage after 34 weeks of gestation’. (C) Phosphorus serum concentration distributions measured between 15^+0^ and 18^+6^ weeks’ gestation, categorized by the pregnancy outcome variable ‘Neonate admitted to the neonatal intensive care unit (NICU)’. Metal elements and pregnancy outcomes included in this figure were selected based on a *P*-value threshold of < 0.001 in logistic regression analyses. For complete results of all logistic regression models, please refer to Table S11.

Manganese (Mn) concentration was positively associated with participants experiencing antepartum haemorrhage (22 participants, 3.2%) after 34 weeks of gestation (OR = 4.62; 95% CI: 2.06–12.4, *P <* 0.001) as was Sr (OR = 1.55; 95% CI: 1.11–2.17, *P =* 0.008). In addition, P concentration was associated with neonates being admitted to the neonatal intensive care unit (NICU; 56 participants; 8.0%) (OR = 2.83; 95% CI: 1.75–4.67, *P <* 0.001) (Fig. 4A–C, S4, S5, and Tables 1, 2, S10, and S11).

**Table 1.** Logistic regression results for the dependent variable ‘Antepartum haemorrhage after 34 weeks of gestation’

	Antepartum haemorrhage after 34 weeks of gestation (ref.: Yes) Total *N =* 683; Ref. *N =* 22; Contrast *N =* 661			
Metal element	OR	95% CI	*P*-value	*q-*value
**Aluminium** (µg/L)	1.39	0.91, 2.01	0.085	0.5
**Antimony** (µg/L)	0.99	0.54, 1.73	>0.9	>0.9
**Barium** (µg/L)	1.17	0.73, 1.72	0.4	0.8
**Boron** (µg/L)	1.03	0.54, 1.78	>0.9	>0.9
**Calcium** (µg/L)	0.75	0.30, 1.79	0.5	0.9
**Chromium** (µg/L)	0.55	0.18, 1.28	0.2	0.6
**Cobalt** (µg/L)	0.37	0.13, 0.89	0.038*	0.4
**Copper** (µg/L)	1.48	0.81, 2.77	0.2	0.6
**Iron** (µg/L)	1.05	0.50, 1.91	0.9	>0.9
**Magnesium** (µg/L)	1.06	0.43, 2.57	>0.9	>0.9
**Manganese** (µg/L)	4.62	2.06, 12.4	<0.001***	**0.027**
**Phosphorus** (µg/L)	1.24	0.45, 3.36	0.7	>0.9
**Potassium** (µg/L)	1.15	0.52, 1.90	0.6	>0.9
**Rubidium** (µg/L)	1.16	0.60, 2.19	0.6	>0.9
**Selenium** (µg/L)	1.58	0.80, 2.69	0.12	0.5
**Strontium** (µg/L)	1.55	1.11, 2.17	0.008**	0.12
**Zinc** (µg/L)	0.59	0.26, 1.23	0.2	0.6

The regression model incorporated 34 independent variables, including the metal elements’ serum concentration, derived from data collected between 15^+0^ and 18^+6^ weeks’ gestation. This table exclusively displays the findings related to metal elements, while the complete set of results can be found in Table S11. OR = Odds Ratio, CI = confidence interval.

*p < 0.05; **p < 0.01; ***p < 0.001.

False discovery rate correction for multiple testing.

**Table 2.** Logistic regression results for the dependent variable ‘Neonate admitted into the neonatal intensive care unit (NICU)’

	Neonate admitted into the neonatal intensive care unit (NICU) (ref.: Yes) Total *N =* 698; Ref. *N =* 56; Contrast *N =* 642			
Metal element	OR	95% CI	*P*-value	*q-*value
**Aluminium** (µg/L)	0.73	0.41, 1.12	0.2	0.6
**Antimony** (µg/L)	0.88	0.62, 1.21	0.4	0.7
**Barium** (µg/L)	0.71	0.41, 1.09	0.2	0.5
**Boron** (µg/L)	0.96	0.64, 1.38	0.8	>0.9
**Calcium** (µg/L)	1.23	0.72, 2.21	0.5	0.7
**Chromium** (µg/L)	0.99	0.52, 1.68	>0.9	>0.9
**Cobalt** (µg/L)	0.89	0.52, 1.35	0.7	0.9
**Copper** (µg/L)	1.13	0.79, 1.60	0.5	0.7
**Iron** (µg/L)	1.29	0.90, 1.77	0.13	0.5
**Magnesium** (µg/L)	0.61	0.39, 0.94	0.029*	0.3
**Manganese** (µg/L)	1.1	0.61, 2.02	0.8	>0.9
**Phosphorus** (µg/L)	2.83	1.75, 4.67	<0.001***	**0.001**
**Potassium** (µg/L)	1.01	0.56, 1.61	>0.9	>0.9
**Rubidium** (µg/L)	0.74	0.48, 1.12	0.2	0.5
**Selenium** (µg/L)	0.63	0.35, 1.09	0.11	0.5
**Strontium** (µg/L)	1.19	0.83, 1.55	0.3	0.7
**Zinc** (µg/L)	0.96	0.65, 1.35	0.8	>0.9

OR = Odds ratio, CI = confidence interval.

*P < 0.05; **P < 0.01; ***P < 0.001.

False discovery rate correction for multiple testing.

## Methods

### Study design and participants

Samples and data utilized in this study were collected as part of UPBEAT (UK Pregnancies Better Eating and Activity Trial), the methods of which have been previously reported [16, 17]. Ethical approval for the study was obtained by the NHS Research Ethics Committee (UK IRAS integrated research application system; reference 09/H0802/5). In brief, UPBEAT was a randomized controlled trial of a behavioural intervention in 1555 pregnant women living with obesity in the UK, which was conducted between March 2009 and June 2014. The intervention aimed to reduce gestational diabetes mellitus (GDM) and large-for-gestational age (LGA) infants by improving diet and physical activity in women living in eight inner city locations across the UK. Women were eligible to be included if they were over 16 years with a body mass index (BMI) ≥ 30 kg/m^2^, had a singleton pregnancy and gestational age between 15^+0^ and 18^+6^ weeks. Women were excluded if they were unwilling or unable to give informed consent, if they had underlying health conditions or were taking metformin. Women allocated to the intervention received advice from a health trainer to reduce dietary glycaemic load and saturated fat intake while increasing physical activity over eight weeks. Women allocated to the control group did not receive any additional information. All women attended antenatal appointments as per local healthcare provision. The outcomes of GDM and LGA were not influenced by the intervention. However, dietary intake and physical activity were improved, while gestational weight gain was reduced in the intervention group [17].

### Data and sample collection

Women who took part in the study provided blood samples at three visits. For this investigation, blood samples taken at baseline (15^+0^–18^+6^ weeks’ gestation) prior to randomization were included. Blood samples were processed, and serum stored at −80°C until analysis.

Social and demographic data collected included maternal age (years), BMI (kg/m^2^), ethnicity (Black, White, Asian, other), parity (nulliparous, multiparous), smoking (smoking during pregnancy, ex-smoker, non-smoker), educational attainment (none, general certificate of secondary education, vocational qualification, A-level, first- or higher degree) and index of multiple deprivation (score from 1, least deprived, to 5, most deprived).

Dietary patterns were identified using factor analysis as previously reported [18]. The dietary pattern ‘Fruit and vegetables’ was characterized by high intakes of bananas, citrus fruit, dried fruit, fresh fruit, green vegetables, pulses, root vegetables, salad vegetables, tropical fruit, and yoghurt. ‘Cultural food’ was characterized by high intakes of red meat, cassava, white meat, rice including pilau, fried or jollof rice, plantain, and fish. ‘Processed food’ was characterized by high intakes of chocolate, crisps, green vegetables, potatoes, processed meat and meat products, root vegetables, squash and fizzy drinks, sugar free squash and fizzy drinks and chips. Lastly, ‘Snacks’ were characterized by high intakes of biscuits, cookies, cakes, pastries, chocolate, full fat cheese, and sweets.

### Sample analysis

Samples were analysed in the London Metallomics Facility at King’s College London. All sample handling procedures were carried out in an ISO class 5 laminar flow bench to minimize trace element contamination. Purified water with a resistivity ≥ 18.2 MΩ cm from a Milli-Q system was used throughout (Merck Millipore). Concentrated HNO_3_ (67%–69% *w/w*) was obtained as trace metal grade acid from Fisher Scientific and trace metal grade 15- and 50-mL polypropylene centrifuge tubes were bought from Elkay Scientific. The following single and multi-element standard solutions were obtained from High-Purity Standards (HPS) and used as calibrants for the analyses of human serum samples: (a) HPS-137-1120-B-100 analytical mixture for trace elements in serum containing Al, As, Be, Cd, Cr, Co, Pb, Ni, Re, Rb, U, V (1000 mg L^−1^) in aqueous 4% HNO_3_; (b) HPS-137-1120-B-100 analytical mixture for trace elements in serum with Sb, B, Hf, Mo, Nb, Ag, Ta, Te, Sn, Ti, W, Zr (1000 mg L^−1^) in aqueous 4% HNO_3_ and 1% HF; (c) HPS 100050-4F-100 single element solution containing Si from (NH_4_)_2_ SiF_6_ (1000 mg L^−1^) in H_2_O; (d) HPS-137-1121-100 analytical mixture for minor elements in serum containing Ba, Cu, Fe, Li, Mn, Se, Sr, S, Zn (1000 mg L^−1^) in 4% HNO_3_; (e) HPS 100039-1-100 single element solution containing P from NH_4_H_2_PO_4_ (1000 mg L^−1^) in 0.05% HNO_3_; and (f) HPS ICP-AM-15-1M-100 analytical mixture for major elements in serum containing Ca, Mg, K, Na (1000 mg L^−1^) in 2% HNO_3_. Single element standard solutions containing 100 mg L^−1^ of Ga, Y, and Ir were obtained from Teledyne Leeman Labs and used as internal standards. The following reference materials were prepared and measured during the analyses to validate the method: Seronorm Trace Elements in Serum L-1 (REF: 201 405, LOT: 1 801 802); HPS CRM Trace Metals in Drinking Water (CRM-TMDW-500); and 125 mL of sterilized human serum obtained from Sigma-Aldrich was employed as an *in*-house serum standard.

### Human serum sample preparation

Frozen human serum samples were allowed to thaw at room temperature for one hour before sample preparation. All samples were prepared in 15 mL centrifuge tubes by diluting 0.15 mL of human serum in 2.85 mL of 0.1 M HNO_3_. Before diluting, the 0.1 M HNO_3_ stock was spiked with Ga, Y and Ir to produce internal standard concentrations of 10 µg L^−1^ in each diluted human serum sample. Calibration standards were made volumetrically in 50-mL centrifuge tubes using single and multi-element standards that were diluted in 0.1 M HNO_3_ and spiked with the same internal standards to obtain a consistent concentration of 10 µg L^−1^ across both calibrants and samples. Calibrant concentrations ranged between 0.01 and 10 µg L^−1^ for trace elements, 1 and 1000 µg L^−1^ for minor elements, and 0.1 and 15 mg L^−1^ for major elements. Blank solutions were made using the same 0.1 M HNO_3_ and internal standard stocks used for both samples and calibrants. Both Seronorm Serum L-1 and the *in*-house human serum standard were prepared in the same manner as the human serum samples; CRM-TMDW-500 was diluted by a factor of 2× and subsequently spiked with the internal standards.

### Determination of element concentrations

All measurements were conducted on a Perkin Elmer NexION 350D Inductively Coupled Plasma Quadrupole Mass Spectrometer (ICP-QMS) operating in both Dynamic Reaction Cell mode (DRC with ammonia gas) and Kinetic Energy Discrimination mode (KED with helium gas). The introduction system to the instrument was a Cetac ASX-520 autosampler coupled to a SeaSpray glass nebulizer that was fitted to a quartz cyclonic spray chamber. The typical settings used for ICP-QMS measurements across the different batches of analysis are presented in the Supplementary data (Table S12). Data reduction involved the normalization of raw intensities using internal standard measurements (Ga, Y, and Ir) and blank correcting this signal by removing the average analyte intensity of blank measurements. Finally, external standardization was applied using a six-point calibration curve to convert the corrected intensities into concentration measurements.

Overall, 887 samples were analysed to determine the serum concentration of Ag, Al, As, B, Ba, Be, Ca, Cd, Co, Cr, Cu, Fe, Hf, K, Li, Mg, Mn, Mo, Na, Nb, Ni, P, Pb, Rb, Re, Sb, Se, Si, Sn, Sr, Ta, Te, Ti, U, V, W, Zn, and Zr. The limit of quantification (LOQ) for each element was calculated using the standard deviation (SD) of blank measurements and the slope of the calibration curve (m), where LOQ = 10 (SD/m). More than 98% of human serum samples exhibited Be, Hf, Nb, Re, Ta, U, W, and Zr concentrations that were below the LOQ and were subsequently excluded from the metallomic analysis. Molybdenum was affected by poor washout throughout the batch runs and was therefore not considered for any further analysis. Quality control solutions consisting of reference materials Seronorm Serum L-1, CRM-TMDW-500, and an *in*-house human serum standard were measured after every 20 samples in each batch of analysis to assess the precision and bias of the method. These data, along with the figures of merit, are provided in Table S13 of the Supplementary data. Importantly, all data produced in this study are identical, within the quoted uncertainties, to the reference values of both Seronorm Serum L-1 and CRM-TMDW-500, except for Ni, which exhibited a higher mean concentration for Seronorm Serum L-1 (9.9 ± 0.9 compared to 5.1 ± 0.9 µg L^−1^; *n* = 43). Any instrument bias was therefore removed by correcting, on a batch-per-batch basis, the mean of the bracketed Seronorm Serum L-1 measurements to the reference value and applying the corresponding correction factor to the respective element concentration of the bracketed sample. For those elements that did not have a Seronorm Serum L-1 reference value, element concentrations were corrected using the same technique but employing the mean concentration of the *in*-house human serum standard measured across the different batches to calculate the correction factor. When there was no Seronorm Serum L-1 reference value available and concentrations for the *in*-house human serum standard were below the LOQ, element concentrations were corrected using the bracketed CRM-TMDW-500 (i.e. As, Ag, Cd, and Te).

### Statistical analysis

#### Data preparation

Data containing participant characteristics collected at baseline were organized and cleaned to facilitate analysis. This pre-processing step involved converting continuous or ordinal variables into categorical variables, grouping similar levels within categorical variables, and simplifying variable or level names to enhance result comprehension and increase participant counts within each category level. A total of 66 variables were selected for statistical analysis, comprising 40 variables collected at study entry and 26 variables during late pregnancy (over 34 weeks of gestation), delivery, and postnatal period. These variables were categorized into 12 groups: ‘Medical Centre’, ‘Registration’, ‘Consent and Eligibility’, ‘Demographics’, ‘Maternal History’, ‘Dietary’, ‘Current Pregnancy’, ‘Maternal Late Pregnancy’, ‘Delivery Data’, ‘Newborn Data’, ‘Neonatal Data’, and ‘End Report’. Further details regarding all metadata variables utilized in the study can be found in Table S1 of the Supplementary Data.

The serum metallomics data were cleaned and organized by excluding participants with duplicated entries or exceptionally low element concentrations across most elements. Additionally, participants were excluded if their data exhibited inconsistencies, problematic samples, or errors during sample preparation. Also, individuals with missing IDs, batch information, or blood sample acquisition dates were excluded. As a result of these cleaning steps, the sample size was reduced from 887 to 881 participants.

Metal elements with more than 120 participants having missing values due to quality control issues, as described in the methods section ‘Determination of element concentrations’, were excluded to maintain the integrity of the analysis and avoid considerably decreasing the number of participants for further analysis. The excluded metal elements were Ag, As, Be, Cd, Hf, Li, Mo, Nb, Ni, Pb, Re, Si, Sn, Ta, Te, Ti, U, V, W, and Zr. Additionally, participants with missing data in any metal element were excluded from the analysis. Consequently, the metallomics dataset comprised data from 755 participants and included 18 metal elements: aluminium (Al), boron (B), barium (Ba), calcium (Ca), cobalt (Co), chromium (Cr), copper (Cu), iron (Fe), potassium (K), magnesium (Mg), manganese (Mn), sodium (Na), phosphorus (P), rubidium (Rb), antimony (Sb), selenium (Se), strontium (Sr), and zinc (Zn).

Finally, early pregnancy, specifically 15–18 weeks’ at which the samples in this study were collected, is associated with increasing plasma volume. Since this can lead to a substantive dilution of water-soluble salts, we assessed whether data adjustment for gestational age was necessary. However, results remained similar and therefore unadjusted results are described.

#### Metallomics data batch correction

The serum samples were processed in six batches that had distinct distributions across medical centres, participants’ living areas and collection dates (Fig. S6A). A visual examination using Uniform Manifold Approximation and Projection (UMAP) revealed that the samples clustered by batch and, to a lesser extent, clustered based on the medical centre of enrolment, underscoring the possible batch effects on the metal elements concentration data (Fig. S6B). A multinomial regression analysis highlighted significant associations between metal element blood concentration and batch, particularly for cobalt, followed by barium and aluminium. Similar analyses were conducted for the medical centre and participants’ living area (Fig. S7A). The batch effects were addressed using the batch effect correction method ‘ComBat’, which adjusts for batch effects using an empirical Bayes framework, from the R package ‘sva’ [19] with parametric adjustment and with the remaining parameters set as default.

After batch correction, we observed that the data samples no longer clustered by batch, and significant associations with batch pre-correction became non-significant post-correction, while some associations with medical centre and participants’ living area remained, suggesting that the batch effect correction was specific for the batch (Fig. S7A). Importantly, the impact of batch effect correction on metal element serum concentration distributions and average values across participants was minimal (Fig. S7B and Table S14).

#### Metal element concentration correlation

The serum concentrations of various elements were analysed for correlation using the ‘cor’ function from the R package ‘stats’, employing the ‘spearman’ method to compute a Spearman correlation coefficient. The remaining parameters were kept as default. Significance testing for these correlations was conducted using the ‘corr_cross’ function from the R package ‘lares’ [20]. This function employs the ‘cor.test’ function from the ‘stats’ package to test the correlation between paired samples, utilizing the Spearman correlation coefficient and a two-sided alternative hypothesis.

To compare the distribution of average scale metal element concentrations among different categorical levels and to assess paired correlations between elements, the Wilcoxon test of significance was employed and adjusted *P*-values were calculated using the Holm method.

The dietary pattern scores were centred around a score of zero. These scores were categorized as ‘Below average’ for scores falling below half of the negative standard deviation and as ‘Above average’ for scores surpassing half of the positive standard deviation.

Regarding the index of multiple deprivation variable, participants were classified as ‘Least deprived’ if they were associated with indices 1, 2, and 3, and as ‘Most deprived’ if they were linked to indices 4 and 5.

#### Adjusted regression analyses

The data collected at baseline were analysed with metal element concentrations serving as the dependent variables and the 37 participant characteristic variables as independent variables in a median regression (Tables S8 and S9). Median regression was chosen due to its robustness to outliers and the observed non-normal distribution and heteroscedasticity of residuals in linear regression models [21]. The median regression was conducted using the ‘rq’ function from the R package ‘quantreg’ [22], employing the Frish–Newton interior point method and with all other parameters maintained at their defaults. Median regression statistics were computed using the ‘summ’ function from the R package ‘jtools’ [23], with standard errors computed through bootstrapping with 1000 bootstrap replications, while the remaining parameters were left at their default values.

Furthermore, a correlation analysis was conducted among all independent variables, and the Variance Inflation Factor (VIF) was calculated to detect potential multicollinearity. The VIF was computed by first performing a linear regression with the same data and variable inputs as the median regression. Subsequently, the ‘vif’ function from the R package ‘car’ [24] was employed, with all parameters set to their defaults. Although Spearman's rho indicated a high correlation between ‘Sleep per night during weekdays’ and ‘Sleep per night during weekends’, as well as between ‘Systolic blood pressure’ and ‘Diastolic blood pressure’ (Fig. S2), the maximum VIF value did not exceed 3.4, suggesting minimal to negligible multicollinearity effects (Table S8). The Akaike information criterion (AIC) was calculated using the ‘AIC.rq’ function from the R package ‘quantreg’ [22], with the median regression model as input and all parameters maintained at their defaults.

The partial residual plots were generated using the ‘effect_plot’ function from the R package ‘jtools’ and by setting the parameter ‘partial.residuals’ to ‘TRUE’ to plot the partial residuals, controlling for the effects of variables besides the independent variable of interest.

It is worth noting that incorporating all 37 participant characteristic variables into the median regression analysis led to a reduction in the participant count from 755 to 531 across all regression models due to missing data on the participant characteristics variables (Table S8).

#### Logistic regression analyses

The logistic regression analyses to determine the association between serum metal concentrations and pregnancy outcomes were conducted to model 26 pregnancy outcome variables by incorporating 17 participant characteristics variables and 17 serum metal element concentrations as independent variables, all obtained during mid-pregnancy. These 17 participant characteristics variables were selected based on their significance in adjusted analyses and their relevance as potential confounders. All numeric variables were scaled prior to the regression analysis. The logistic regression was performed using the ‘glm’ function from the R package ‘stats’ [25], employing a binomial error distribution and a ‘logit’ link function. All remaining parameters were kept at their default settings. The model statistics were calculated using the ‘summary’ function from base R. The *P*-value and *q-*value were calculated using the R package ‘gtsummary’ [26], where the *P*-value was obtained from a Wilcoxon test for continuous variables and a chi-square test for categorical variables. The q-value was calculated using the Benjamini & Hochberg method [27] (Tables S10 and S11).

To detect multicollinearity, a correlation analysis was performed among all independent variables and the VIF was calculated (Fig. S4 and Table S10). The VIF calculation was performed by first conducting a linear model on the same data and formula as the logistic regression, with the model then used as input for the ‘vif’ function from the R package ‘car’ [24]. As observed in Fig. 2, a high Spearman's correlation coefficient of 0.82 was exhibited between the serum concentration of calcium and sodium. Additionally, including both calcium and sodium as independent variables in the logistic regression models resulted in VIF values exceeding 5, leading to the exclusion of sodium from further analysis. Table S10 provides information on the dependent variable, number of participants, AIC, residual deviance, maximum VIF, and the variable responsible for the maximum VIF for each regression analysis.

## Discussion

In this study, we investigated the relationship between serum metal element concentrations in pregnant women with obesity and various maternal demographic characteristics, as well as pregnancy outcomes. To our knowledge, this is the first study to assess the metallomic profile of this high-risk population, providing a comprehensive dataset that serves as a foundation for future research. Notably, our findings suggest associations between serum metal element concentrations and factors such as age, dietary patterns, education, BMI, parity, and metabolic markers. Na displayed the highest mean concentration at 175.2 mmol/L (4027.73 mg/L), which is above the reference non-pregnancy serum range of 137–142 mmol/L [28, 29]. The higher values observed in our study likely reflect methodological differences, as inductively coupled plasma mass spectrometry measures the total sodium content in serum samples. Our analysis has therefore been influenced by various sodium salts in the blood, predominantly sodium bicarbonate and sodium phosphate, as well as protein-bound sodium, which may explain these elevated concentration values [30, 31]. Notably, in our analysis, participants residing outside urban areas had lower Na concentrations compared to those living in inner-city environments, even after adjusting for variables such as dietary patterns and BMI. The mean serum Cu concentration was 2322 µg/L, exceeding the non-pregnancy reference range of 635–1589 µg/L [32]. However, it remained within a previously reported reference range for pregnant women, 1070–3624 µg/L [33]. During pregnancy, Cu levels in maternal serum rise in parallel with ceruloplasmin, with total body Cu levels increasing [34, 35]. Our results revealed a positive correlation between Cu and BMI, consistent with previous research linking elevated serum Cu levels to obesity [36]. This positive correlation likely reflects the association of elevated serum Cu levels with obesity-related inflammation and altered copper metabolism [35]. Cu was the element with the strongest positive correlation with BMI, while all other elements showed low or non-significant correlations with serum concentrations as BMI increased above 35 kg/m², likely due to a higher blood volume distribution. The mean Ca concentration was 106.37 mg/L (2.65 mmol/L), slightly exceeding the upper limit of the non-pregnancy reference range (2.1 to 2.6 mmol/L) [37, 38]. During pregnancy, women typically exhibit higher circulating serum Ca compared to non-pregnant women due to increased demand for fetal development [39]. However, our findings suggest that parity may influence Ca homeostasis, as previously observed [40], with both primiparous and multiparous participants showing lower concentrations compared to nulliparous participants. Multiparity has been linked to reduced bone mineral density in postmenopausal women, with a possible increased risk of lumbar osteoporotic fractures [41]. Although the participants in our study were not of postmenopausal age, this association highlights the potential impact of parity on bone density later in life. Additionally, variations in vitamin D status, which may differ across ethnic groups, could influence this relationship [42, 43]. While our analysis did not include vitamin D status, it did account for ethnicity. Nevertheless, the absence of vitamin D data remains a limitation of this study. Additionally, our analysis also highlighted associations between metal elements including Ca and Na. High salt intake is considered a risk factor for osteoporosis, inducing calcinuria and affecting bone calcium balance [44, 45]. In our analyses, we observed a positive association between the participant’s age and the concentration of B, Fe, and Se, suggesting that age may be an important factor when assessing the impact of metal exposure on gestational health and outcomes [46–50]. Additionally, we noted that the participant’s geographical location and dietary patterns were associated with the overall blood metal levels, indicating that metal homeostasis during pregnancy is influenced by both physiological changes and nutritional status. Diet quality had a notable effect on metal concentrations, with Se and B, which have documented health benefits [51–53], being higher in participants who consumed more fruits, vegetables, and cultural foods, and lower in those with diets high in processed foods and snacks. We also observed significantly higher Ba concentrations in women with obesity from suburban, town, or rural areas, also among those with lower processed food and snacks intake, suggesting that environmental factors or associated diet may play a role [54]. Further research is needed to explore the health implications of elevated Ba concentrations during pregnancy associated with obesity [55]. We observed an association between B and a lower ratio of LDL to HDL in the blood, as previously reported [56]. Higher plasma B concentrations have been linked to a healthier diet, lower BMI, and a more favourable cardio-metabolic risk profile [57]. However, in our data, B was not associated with BMI, possibly due to the already high BMI of the participant group or higher volume distribution. Nonetheless, B was positively associated with consumption of fruits and vegetables. The observed positive correlation between P and cholesterol levels in this cohort of women living with obesity is in line with prior research in non-pregnant populations. Earlier studies have indicated a relationship between phosphate and campesterol levels in individuals with kidney failure, suggesting a connection between phosphate and cholesterol metabolism that could influence cardiovascular outcomes [58]. However, it is important to note that in our analysis, we measured phosphorus concentration, which differs from the parameters typically measured in clinical settings, such as serum phosphate concentration. In our study, elevated maternal P levels were associated with admissions to the NICU, implying a potential impact on fetal development and neonatal health [59]. Further investigation is necessary to fully comprehend the mechanisms and consequences of high phosphorus levels during pregnancy. Elevated concentration of Mn was linked to antepartum haemorrhage, emphasizing the potential significance of this metal in vascular health and coagulation [60–62]. The cohort experiencing antepartum haemorrhage exhibited a median Mn level of 8.1 µg/L, while the non-affected group displayed a median value of 4.8 µg/L. Further investigation is required to understand the correlation between serum concentrations of Mn and antepartum haemorrhage. Whilst our study offers valuable insights into the metallomic profiles of pregnant women with obesity, several limitations should be acknowledged. Firstly, the observational nature of the study limits our ability to establish causality between metal element concentrations and pregnancy outcomes. Therefore, the statistical associations identified in this study should be interpreted as correlations rather than causal relationships. Additionally, the reliance on self-reported data for certain variables, such as dietary patterns, introduces the potential for recall bias. The generalizability of our findings may be limited to populations with similar demographic characteristics to those included in the UPBEAT trial, and the complexity of pregnancy and its outcomes necessitates consideration of multifactorial influences beyond the variables considered in this study. Furthermore, due to the original study design of the UPBEAT study and a lack of a suitable comparator population, our analysis is limited to participants with a BMI above 30 kg/m², without including individuals with lower BMIs or non-pregnant women for comparison. Finally, the association between elevated maternal P levels and NICU admission should be interpreted cautiously, as NICU admission is influenced by various factors that were not fully captured in our analysis and may confound the observed relationship.

## Conclusion

Our study provides new insights into the complex interplay between metal element concentrations, maternal characteristics, and pregnancy outcomes. The observed associations underscore the importance of further research to unravel the biological mechanisms underlying these relationships. Such efforts are essential to inform potential interventions aimed at optimizing maternal and neonatal health outcomes in pregnancy. Improving our understanding of the relationships between metals and maternal health has significant public health potential, as it can inform policies aimed at reducing pregnancy-related risks and supporting healthier pregnancies.

## Supplementary Material

mfaf031_Supplemental_Files

## Data Availability

The data underlying this paper cannot be shared publicly to protect the privacy of individuals who participated in the clinical study. Access to the data may be granted upon reasonable request to the corresponding author and in accordance with ethical and institutional guidelines. The code used for data analysis is available at https://github.com/vonMeyennLab/UPBEAT-metallomics [DOI: 10.5281/zenodo.16742673].
