# Supplementary material for: Metallomic profiles of pregnant women living with obesity in the UK: a secondary analysis of UPBEAT
Source: Metallomics. 2025 Aug 12;17(8):mfaf031. doi: 10.1093/mtomcs/mfaf031 (PMC12378400; doi:10.1093/mtomcs/mfaf031)
Supplement: mfaf031_Supplemental_Files [file mfaf031_supplemental_files.zip › suppl_data_table_legend_revised.docx]

# **Supplementary tables**

**Table S1.** List of continuous and categorical metadata variables used in the study. The table displays the number and percentage of participants in each category level for categorical variables and the median and interquartile range (IQR) values for continuous variables. The variables are organized into distinct categories: Medical Centre, Registration, Consent and Eligibility, Demography, Maternal History, Current Pregnancy, Dietary, Maternal Late Pregnancy, Delivery Data, Neonatal Data, and End Report. This arrangement, coupled with a concise description, is included to enhance comprehension of individual variables. After evaluating the need for gestational age adjustment, only unadjusted results are reported.

**Table S2.** Spearman's rank correlation coefficient, also referred to as Spearman's rho, of the correlation between all paired combinations of metal elements. These correlation coefficients were calculated using the metal element’s serum concentrations.

**Table S3.** Average scaled serum concentration values for each metal element across a subset of participant characteristics. The subset comprises age, ethnicity, body mass index, living area, index of multiple deprivation, parity, and dietary patterns categorized by consumption of fruits and vegetables, cultural food, processed food, and snacks. These values denote the mean serum concentration of each metal element averaged across all 755 participants.

**Table S4.** Statistical significance testing results for the average scaled serum concentration of metal elements across categorical levels of the variables presented in Figure 2D and Table S3. The p-values were computed using a Wilcoxon test. The adjusted p-value was calculated using the Holm method. *p < 0.05; **p < 0.01; ***p < 0.001.

**Table S5.** Comparison of the mean serum concentrations of each element across the categorical levels of the variables presented in Figure 2D and Table S3. The p-values were computed using a Wilcoxon test. The adjusted p-value was calculated using the Holm method. *p < 0.05; **p < 0.01; ***p < 0.001.

**Table S6.** Spearman's rank correlation coefficient, commonly known as Spearman's rho, of the correlation between paired combinations of elements with a p-value below 0.05. These correlation coefficients were calculated using the metal element’s serum concentrations. The results are presented for a subset of participant characteristics, including age, ethnicity, body mass index, living area, index of multiple deprivation, parity, and dietary patterns categorized by the consumption of fruits and vegetables, cultural food, processed food, and snacks.

**Table S7.** Statistical significance testing results for the correlation between the serum concentration of metal elements across categorical levels of the variables depicted in Figure 2E and Table S6. The p-values were computed using the 'corr_cross' function from the 'lares' R package. The adjusted p-value was calculated using the Holm method. *p < 0.05; **p < 0.01; ***p < 0.001.

**Table S8.** Statistical metrics derived from the median regression analysis results presented in Figure 3 and Table S9, where each metal elements’ serum concentration serves as the dependent variable and the 37 metadata variables collected between 15^+0^ to 18^+6^ weeks' gestation as the independent variables. The table includes the number of participants used in the model, the Akaike information criterion (AIC), the Bayesian information criterion (BIC), the model fit denoted by an R value, and the maximum Generalized Variance Inflation Factor (GVIF) value for each model, assessing the extent of multicollinearity among independent variables.

**Table S9.** Results from the median regression analysis with each metal elements’ serum concentration as the dependent variable and 37 metadata variables collected between 15^+0^ to 18^+6^ weeks' gestation as the independent variables. The table provides the estimates and 95% confidence intervals (CIs) for each variable and categorical level. Additionally, it presents the statistical significance (p-value) and the number of participants associated with each variable or categorical level. *p < 0.05; **p < 0.01; ***p < 0.001.

**Table S10.** Statistical metrics derived from the logistic regression analyses presented in Table S11, where each of the 26 pregnancy outcome variables served as the dependent variable in individual models. The models included 17 metadata variables collected between 15^+0^ to 18^+6^ weeks' gestation and 17 metal elements' serum concentrations as independent variables. Sodium was excluded due to high correlation with Calcium (refer to the Methods section for further details). The table includes the number of participants used in each model, the Akaike information criterion (AIC), residual deviance, the maximum Generalized Variance Inflation Factor (GVIF) value for each model, assessing the extent of multicollinearity among independent variables, and the variable displaying the maximum GVIF value.

**Table S11.** Results from the logistic regression analysis, where each of the 26 pregnancy outcome variables served as the dependent variable in individual models. The models included 17 metadata variables collected between 15^+0^ to 18^+6^ weeks' gestation and 17 metal elements' serum concentrations as independent variables. Sodium was excluded due to high correlation with Calcium (refer to the Methods section for further details). The table provides information about the total number of participants included in the model, participants in the reference categorical level, participants in the contrast categorical level, odds ratio (OR), 95% confidence interval (CI), statistical significance, and q-value estimating false discovery rate (FDR). A positive odds ratio indicates a positive association between the variable (or variable categorical level) and the reference level of the pregnancy outcome variable, the independent variable in the model. The p-value and q-value were calculated using the R package 'gtsummary', where the p-value was derived from a Wilcoxon test for continuous variables and a chi-square test for categorical variables, and the q-value was calculated using the Benjamini & Hochberg method. *p < 0.05; **p < 0.01; ***p < 0.001.

**Table S12.** Instrument and method acquisition parameters of the ICP-QMS used for element concentration measurements of human serum samples.

**Table S13.** Method validation and element concentrations determined for reference materials Seronorm Trace Elements in Serum L-1, HPS CRM-TMDW-500, and an *in*-house human serum standard all of which were acquired during multiple batches of human serum sample analyses.

^a^ Mean r^2^ value determined from *n* independent calibration curves during the analysis of different batches of human serum samples. ^b^ Mean limit of quantification calculated from the different batches of analyses and based on a total of *n* individual blank measurements. If the value is displayed as 0.00 μg L^-1^, the LOQ for the given element is < 10 ng L^-1^. ^c^ Mean element concentrations for each reference material determined from *n* individual sample aliquots prepared for ICP-QMS analysis. ^d^ 2 x standard deviation (2SD) calculated from individual results for *n* sample aliquots analysed across several batches of measurements (i.e., long-term reproducibility). ^e^ Relative standard deviation (2RSD) as a percentage of the absolute concentration for a given element. ^f^ Deviation of the measured element concentration from the given reference value as a percentage of the absolute concentration.

**Table S14.** Median and interquartile range (IQR) of serum concentrations for each metal element before and after batch correction, calculated from the metallomics data of all 755 participants.
